# Etiology and Prevention of Perineal Lacerations, with Special Reference to the Shoulders

**Published:** 1905-10

**Authors:** Archibald Smith

**Affiliations:** Atlanta, Ga.


### ETIOLOGY AND PREVENTION OF PERINEAL LACERATIONS, WITH SPECIAL REFERENCE TO THE SHOULDERS.

By ARCHIBALD SMITH, M. D., Atlanta.

Perineal lacerations of ordinary degree, though not usually considered serious complications of labor, are perhaps, on the whole, the most troublesome that we have to deal with.

The great trouble caused by them is due in part to their frequency, and also to the fact that an open wound in this locality, especially if not properly treated, greatly increases the danger of sepsis, not to mention the increased liability to displacements of the pelvic viscera. The frequency of perineal lacerations has been stated as ranging all the way from thirty-five per cent. in primipara and ten per cent. in multipara, in some of the large maternity clinics, down to one per cent., or even none, in the practice of some men who claim to have delivered large numbers of women, but probably never had any hospital experience.

Two comments might be made, in passing, on the latter statement—first, that it is not based on the observation of facts, and second, that it is largely from the patients of such men that the gynecologist derives his perineorraphies, and Lydia E. Pinkham her customers.

The proper handling of the perineum, though much less spectacular in appearance, is, nevertheless, more important to the average practitioner than the performance of a Cæsarean section, for he will seldom encounter a case where the latter is required, and when he does he will, as a rule, find it both practicable and advisable to summon the aid of a man skilled in abdominal surgery. But he will have an opportunity to show his skill or lack of skill in handling the perineum at almost every labor he may attend.

Briefly and in general terms, lacerations of the perineum may be said to be due to the fact that the parts of the child are larger than the vulvar orifice through which they must pass, and in order that passage may take place, the tissues surrounding this orifice must be either greatly stretched or sustain a solution of continuity.

Therefore, in avoiding perineal lacerations, we must, as far as possible, bring the smallest diameters of the child to coincide with those of the pelvic outlet during their passage through it, avoid direct impact of abrupt parts, like shoulders, against the perineum, and last, but not least, allow ample time for enlargement of the vulvar orifice to take place by stretching instead of tearing.

In delivering the head all of these points must be taken into consideration, and the anterior positions of the occiput are most favorable to them; when a posterior position is diagnosed before engagement of the head an attempt should be made to convert it into an anterior position by external manipulation and postural treatment.

Now suppose the head to be at the perineum in the L. O. A. position, and making the usual progress, our chief concern will be to regulate its rate of progress and degree of flexion.

When a pain occurs the head should be allowed to advance till the perineum is well stretched, and then firmly held in position with the thumb and forefinger till the pain passes off. If after this the perineum remains thin and white, the head should be pushed back enough to allow reestablishment of the circulation, which keeps up the vitality and elasticity of the parts, thus enabling them to stand greater dilation. An anesthetic may be used here with great advantage, both to control the contractions and relieve the suffering of the patient. If an anesthetic is not thought advisable, or before it is begun, the old method of making the patient open the mouth and breathe rapidly, during a pain, will greatly assist in regulating the progress of the head.

Some advise crowding the head up under the symphysis, but while this gets the occiput away from the perineum it also is apt to produce extension too soon and bring the head out by some of its larger diameters. It is well to allow the occiput to extend and even to push it forward during the last few pains, but the nape of the neck should appear well under the symphysis and the anterior fontanel, over the perineum, before any marked extension is brought about. When the head has reached this point it should be delivered between the pains, by extension, keeping the suboccipital region pressed well against the symphysis and bringing it out of the vulva by a motion of extension and rotation on this as a fixed point. This motion may be brought about by forward pressure on the vertex, and also pressure with the fingers through the perineum exerted against the brows, cheeks and chin, but in this care must be taken not to injure the nose or eyes.

The rationale of this method will be apparent if we will consider, for a moment, the diameters of the head, which must pass through the vulva by this motion, the largest of which is the S. O. F. of 10.50 cm. against the O. F. of 11.75 and O. M. of 13.50 cm. if the head came out in full extension, with the occiput under the symphysis as the chin or frontal region passed the perineum. It is also evident that if partial extension occurs the strain will be greater according as the point under the symphysis approaches the occiput.

Even when the head engages with the occiput posterior it will generally undergo spontaneous anterior rotation, but if this does not occur it can be often brought about by inserting the single blade of the forceps into the vagina to furnish resistance, or by using the fingers in the same manner, and at the same time pressing forward on the occiput. External pressure may also be tried by placing the fingers on the perineum in the region of the anus, and on the side opposite that to which the head should rotate.

### PERSISTENT POSTERIOR POSITIONS OF THE OCCIPUT.

When these means fail to produce anterior rotation, the use of forceps in skilled hands may aid in preserving the perineum either by directly rotating the head or by lifting it over the perineum. If improperly applied, however, they are capable of doing great damage to both mother and child, and should be avoided if possible.

Birth of the head in a posterior position may occur by one or two similar mechanisms, though the effect on the perineum is quite different. First and most favorable, a point a little anterior to the large fontanel strikes the symphysis and the posterior part of the head is elongated, and gradually comes out over the perineum to the sub-occipital region, after which the head is extended and pressed back on the perineum while the face appears under the symphysis.

In the second and, fortunately, less common mechanism there is greater extension and the base of the nose impinges against the symphysis instead of the point near the fontanel, otherwise the mechanism is much the same, except that the O. F. diameter 11.75 must pass the vulva instead of the T. O. F. diameter of 10.50 cm., thereby greatly increasing the difficulty of delivery and danger of laceration.

These positions should be carefully looked out for, and when diagnosed great care should be taken to maintain a good degree of flexion by inserting the fingers behind the occiput and pressing forward, also by retarding as much as possible the advance of the frontal region, which may also be pressed against the symphysis with advantage, just as the anterior fontanel appears. This position should be maintained if possible till the nape of the neck appears over the perineum, when the face may be born much as in an anterior position of the occiput, except that the head rotates around the sub-occipital, which is resting on the perineum instead of the symphysis.

The prognosis for the perineum in breech presentations is not favorable because the soft body does not thoroughly dilate it, and when the head reaches this point it must be delivered at once, without allowing further time for dilatation. A child presenting by the breech should always be brought down with the dorsum anterior if possible, so as to prevent the chin from catching under the symphysis.

When the occiput is anterior, if we bear in mind the various diameters of the head, we will readily see that the same principles may be applied as in delivering the vertex, namely, to keep the head well flexed till the sub-occipital region appears under the symphysis, and then deliver the features from under the perineum in reverse order. This is the best done by Wigand's or Moriceau's method, both of which keep the head flexed by the forefinger in the mouth. The former is usually preferable, as the pressure on the head with the free hand aids in maintaining flexion, while the free hand in Moriceau's method grasps the shoulders and makes traction on the neck. If this, however, is supplemented by pressure on the head by an assistant it is most efficient of all.

It the occiput comes down posteriorly it will usually be necessary to raise the body of the child and rotate the head and neck from under the symphysis on a point near the larynx. This requires great care not to injure the child by too firm pressure on the larynx, and it is better to transgress a little on the perineum than on the throat of the child.

In cases where it becomes evident that the vulvar orifice will not enlarge enough to admit the passage of the head without a solution of continuity of surrounding tissues, the well-known operation of episiotomy may be performed. This has the advantage of substituting for the perineal laceration a clean-cut incision on either side of the vulva, which can be easily repaired, divides less important structures and is less in the way of infection.

THE SHOULDERS.

Many writers on obstetrics pass over the question of delivering the shoulders with a few lines, some simply directing that the head should be supported by the hand and the shoulders allowed to take care of themselves, while others even say that the old writers laid too much stress on them as a factor in perineal tears. However that may be, it seems to me that the pendulum has swung too far in the opposite direction, and the matter does not now receive the attention which it merits. Some observation has led me to believe that more lacerations are either caused, or greatly augumented, by the shoulders than is generally taught. Of course, satisfactory examination of the perineum can not be made between the delivery of the head and shoulders, but if one will notice as carefully as possible the condition of the perineum, just after the head is delivered, and then when delivery is complete, he will find greater increase in the size and number of tears than can be accounted for by better opportunity for observation.

The student is usually advised against dragging the shoulders through the vulva after the head has been born, on account of injuring the perineum, and with this I heartily concur, but, nevertheless, my opinion, after some experience, leads me to believe that more or less active treatment, judiciously applied at this stage, is very advantageous, and will reduce the number and degree of perineal lacerations to a considerable extent.

Delivery of the shoulders may be considered under three heads, according to whether both arms are extended along the sides of the chest, one flexed across the chest with the hand in the region of the neck and the other extended, or both arms flexed with the hands near the neck. When both arms lie extended the delivery of the shoulders is easier than in the other positions, and less liable to cause laceration if left to nature, because we do not hav the size of the hands or forearms added to that of the shoulders during their passage through the vulva.

An advanced position of the hands and forearms can usually be diagnosed when the fingers are passed around the neck in search of the cord, or by inserting the finger along the child's chest after the cord has been removed from the neck, or may at times be visible before the birth of the shoulders. If the forearms are not felt across the chest the posterior shoulder can, as a rule, be most easily delivered first. When an anesthetic is not used this should, if possible, be done between the pains by pressing the neck lightly against the symphysis, and the anterior shoulder upward and backward behind the symphysis, thus retarding its progress till the posterior shoulder is born. This proceeding will carry the posterior shoulder forward and cause it to impinge much less directly on the perineum, and it will at times slip from under it without further assistance, but if this does not occur, the forefinger may be inserted into the vulva, the arm and shoulder carried forward, and then grasped with the thumb and finger and lifted over the perineums. Hooking the finger into the axilla is not generally advisable, as there is danger of injuring the structures in the axilla, and besides it tends to push the point of the shoulder further out against the perineum. If this is done it should be from behind and the force should be chiefly against the thorax. Care must also be taken in grasping the shoulder not to cause too much pressure and fracture the clavicle. This method has the disadvantage of adding the thumb and finger to the bodies which must pass through the vulva, but this, I believe, is more than counterbalanced by the fact that the shoulder is lifted up and the perineum pushed away from it.

If the anterior shoulder does not catch on the symphysis and is coming down first, the posterior one should be carefully retarded by holding it back with one hand and exerting a careful downward traction on the neck so as to deliver the anterior shoulder first, then after pressing the arm and shoulder a little forward, so that they shall not come directly under the symphysis, the body of the child is brought well up against the symphysis and the posterior shoulder handled much the same as in delivering it first, or supported by the hand placed over the perineum.

When the anterior arm is flexed and the posterior extended it will usually be best to deliver the anterior shoulder and arm first, when the progress of the posterior shoulder can be arrested without undue force. This can usually be done by a moderate backward and downward pressure on the posterior shoulder and neck, which at the same time should disengage the anterior shoulder from under the symphysis and permit of its delivery. The posterior shoulder should still be held back and the anterior arm and forearm delivered by forward pressure on the former and traction on the latter. If the posterior shoulder is now released it will generally be born spontaneously, but if not can be delivered by raising the head and neck so as to bring the thorax well against the symphysis, and, if necessary, grasping the shoulder as directed above. These manipulations must be done between the pains, for sudden delivery with a sharp pain would almost certainly cause laceration, especially as the elbow was being born. The perineum may be supported here by forward pressure applied over its surface with the outstretched hand, as directed sometimes for the head. The objections against this do not hold good here, for the hand is a real support, inclining the shoulder upward and forward as it should go, and preventing the tendency it has to plow through the perineum; besides, this pressure for the length of time the shoulder requires to be born does not seriously interfere with the circulation of the perineum or reduce its vitality.

If the anterior shoulder can not be delivered first, due to engagement under the symphysis and rapid progress of the posterior shoulder, the same method of procedure may be followed as outlined for delivery of the posterior shoulder first with both arms extended.

Where the anterior arm is extended and the posterior flexed it will usually be wise to deliver the posterior shoulder first, much as may be done when both arms are extended, and then deliver the corresponding arm. This allows the hand and arm to be born along with what might be called the cervico-acromial diameter, extending from the base of the neck on one side to the shoulder on the other. This will obviously cause less strain if carefully done than for the hand and arm to be born at the same time as the bisacromial diameter, which would be the case if the anterior shoulder were delivered first. If any difficulty is experienced in delivering the posterior arm before the anterior shoulder it should be left alone, for the abrupt point of the shoulder has now passed the perineum and the pressure exerted on it is by the soft tissues of the arm and thorax.

If there is a strong tendency for the anterior shoulder to proceed ahead of the posterior, it may be well to adopt the method of procedure suggested for the same condition when both arms are extended.

When both arms are flexed across the chest we have the condition most likely to cause laceration if left alone, for the perineum is likely to be distended by both shoulders and arms at the same time. About the most favorable thing that can happen at this time is for the anterior shoulder to be disengaged from the symphysis and delivered ahead of the posterior, after which the anterior arm may be delivered, thus allowing the anterior side of the thorax to come in contact with the symphysis and sub-pubic ligament, and relieving the perineum of considerable strain and allowing the posterior shoulder to be delivered in a manner already described.

When the anterior shoulder is impacted on the symphysis and the posterior shows a strong tendency to advance, the same method of procedure may be adopted as when the anterior arm is extended, the posterior flexed and the anterior shoulder is caught under the symphysis.

